# Detection and full genome characterization of two beta CoV viruses related to Middle East respiratory syndrome from bats in Italy

**DOI:** 10.1186/s12985-017-0907-1

**Published:** 2017-12-19

**Authors:** Ana Moreno, Davide Lelli, Luca de Sabato, Guendalina Zaccaria, Arianna Boni, Enrica Sozzi, Alice Prosperi, Antonio Lavazza, Eleonora Cella, Maria Rita Castrucci, Massimo Cicozzi, Gabriele Vaccari

**Affiliations:** 1 Department of Virology, Istituto Zooprofilattico Sperimentale Lombardia ed Emilia Romagna, Via Antonio Bianchi 9, 25124 Brescia, Italy; 2 Department of Food Safety, Istituto Superiore di Sanità. Nutrition and Veterinary Public Health, Viale Regina Elena 299, 00161 Rome, Italy; 3 Dept. of Sciences, University Roma Tre, Viale Guglielmo Marconi 446, 00146 Rome, Italy; 4 University Campus Bio-Medico of Rome, Via Álvaro del Portillo, 21, 00128 Rome, Italy

**Keywords:** MERS-like Beta-CoV viruses, Full genome sequencing, Bats, Italy, Phylogenetic and molecular analyses

## Abstract

**Background:**

Middle East respiratory syndrome coronavirus (MERS-CoV), which belongs to beta group of coronavirus, can infect multiple host species and causes severe diseases in humans. Multiple surveillance and phylogenetic studies suggest a bat origin. In this study, we describe the detection and full genome characterization of two CoVs closely related to MERS-CoV from two Italian bats, *Pipistrellus kuhlii* and Hypsugo savii.

**Methods:**

Pool of viscera were tested by a pan-coronavirus RT-PCR. Virus isolation was attempted by inoculation in different cell lines. Full genome sequencing was performed using the Ion Torrent platform and phylogenetic trees were performed using IQtree software. Similarity plots of CoV clade c genomes were generated by using SSE v1.2. The three dimensional macromolecular structure (3DMMS) of the receptor binding domain (RBD) in the S protein was predicted by sequence-homology method using the protein data bank (PDB).

**Results:**

Both samples resulted positive to the pan-coronavirus RT-PCR (IT-batCoVs) and their genome organization showed identical pattern of MERS CoV. Phylogenetic analysis showed a monophyletic group placed in the Beta2c clade formed by MERS-CoV sequences originating from humans and camels and bat-related sequences from Africa, Italy and China. The comparison of the secondary and 3DMMS of the RBD of IT-batCoVs with MERS, HKU4 and HKU5 bat sequences showed two aa deletions located in a region corresponding to the external subdomain of MERS-RBD in IT-batCoV and HKU5 RBDs.

**Conclusions:**

This study reported two beta CoVs closely related to MERS that were obtained from two bats belonging to two commonly recorded species in Italy (*P. kuhlii* and H. savii). The analysis of the RBD showed similar structure in IT-batCoVs and HKU5 respect to HKU4 sequences. Since the RBD domain of HKU4 but not HKU5 can bind to the human DPP4 receptor for MERS-CoV, it is possible to suggest also for IT-batCoVs the absence of DPP4-binding potential. More surveillance studies are needed to better investigate the potential intermediate hosts that may play a role in the interspecies transmission of known and currently unknown coronaviruses with particular attention to the S protein and the receptor specificity and binding affinity.

## Background

Since the early 70s, a variety of pathological conditions in domestic and wild animals have been attributed to coronavirus (CoV) infections. Currently, six different CoV strains are known to infect humans [1]. Two of these belong to the beta CoV genus, severe acute respiratory syndrome coronavirus (SARS-CoV) and Middle East respiratory syndrome coronavirus (MERS-CoV), and they cause severe respiratory diseases with case fatality rates of 9% and 35%, respectively [2]. The reservoir of these viruses is usually animal with occasional spillover into humans, possibly through an intermediate host species. Apart from animal to human transmission, human-to-human transmission of SARS-CoV and MERS-CoV occurs mainly through nosocomial transmission [3]. Bats, with their extensive geographical distribution and flight capability, have been documented as natural hosts of a large number of diverse viruses, such as lyssaviruses, paramyxoviruses and filoviruses. Moreover, the genetic diversity of CoVs in bats exceeds that known for other hosts, which is compatible with bats being the major reservoir of mammalian CoVs [4].

The evolutionary origin of SARS-CoV, which was first detected in 2002, involved bat hosts, possibly with civets as intermediate host and the source of human infection [4]. The origin of MERS-CoV is not well known, but more recent studies point to camels as possible reservoirs or intermediate hosts. Bats have also been suspected as the evolutionary source of MERS-CoV due to the genetic similarities between beta CoVs found in bats and the MERS-CoV in humans [5, 6].

The receptor binding of CoV is mediated by the Spike protein (S), which is further cleaved into S1 and S2 subunits that are involved in engaging receptors and mediating membrane fusion, respectively. The peptidase recognized by MERS-CoV was identified as dipeptidyl peptidase 4 (DPP4 or CD26) [7]. The S1 domain responsible for the recognition of DPP4 receptor is located in a C-terminal 240-residue receptor binding domain and is composed of a core and an external subdomain. This external subdomain, also designated as the receptor binding motif, engages the receptor. Investigation of the DPP4-binding potential of bat CoVs is essential to better understanding the biology of these viruses, the eventual role in the evolutionary pathway of MERS-CoV and their potential threat to human health. Although high sequence identity in S protein was observed between BatCoVs HKU4/HKU5 and MERS-CoV, it was recently demonstrated that only the RBD of HKU4 was able to bind the human receptor DPP4 [8]. Even if it is less adapted than MERS-RBD and shows lower affinity for receptor binding, the ability of HKU4 to bind human DPP4 indicates its potential for adaptation to infect humans.

On the other hand, other authors [9] reported that MERS-RBD interacts efficiently with Jamaican fruit bat DPP4 receptor and MERS-CoV replicates efficiently in Jamaican fruit bat cells, suggesting that there is no restriction at the receptor or cellular level for MERS-CoV.

A variety of closely MERS-related CoV sequences have been obtained from numerous bat species in different continents. A fragment of a CoV showing 100% identity to HCoV-EMC/2012 cloned from the index MERS case was found in a faecal sample from an Egyptian tomb bat (*Taphozous perforatus*) in Bisha, South Arabia [5]. Partial genome sequences from viruses closely related to MERS-CoV have also been detected in bats from Africa, America and Europe [10–13]. CoVs originated from bats in Africa [6, 14], and in China [15], they were fully sequenced and identified as highly related to MERS-CoV. Despite all these reports, only three regarded the complete genome [6, 14, 15]. The other ones were based on short genomic sequences of a conserved fragment codifying the RNA-dependent RNA polymerase (RdRp) gene, which is less informative and unsuitable for solid phylogenetic hypothesis. In this study, we describe the detection and full genome characterization of two CoVs closely related to MERS-CoV from Italian bats of different species, *Pipistrellus kuhlii* and *Hypsugo savii*.

## Methods

### Sampling

Fresh carcasses of spontaneously dead bats were obtained from a wildlife rehabilitation centre in the context of a virological survey implemented in Northern Italy since 2010. The identification of bat species was made according to morphologic characteristics reported in the illustrated identification key to the bats of Europe [16].

### Pan-coronavirus RT-PCR

Pools of viscera (lung, heart, spleen and liver) and intestine were homogenized in minimal essential medium (MEM, 1 g/10 ml) containing antibiotics and clarified by centrifugation at 3000×g for 15 min. Viral RNA was extracted from 100 μl of sample using the NucleoMag 96 Virus kit (Macherey-Nagel, Düren, Germany). The RNA was eluted in 100 μl of MV6 elution buffer and stored at −80 °C. CoV screening was performed by a pan-coronavirus one-step RT-PCR method based on degenerate primers that amplified a fragment (180 bp) of the RNA-dependent RNA polymerase (RdRp) gene [12].

### Virus isolation attempts

Virus isolation was attempted by inoculation with tissue samples of different cell lines such as VERO cells (African green monkey kidney), MARC-145 (foetal monkey kidney), HRT-18 (human colorectal adenocarcinoma), FRhK 4 (foetal rhesus kidney), LLC-Mk2 (rhesus monkey kidney) and TB1 LU (lung, Mexican free-tailed bat, “*Tadarida brasiliensis* mexicana”). Confluent monolayers of cell lines were inoculated with samples, incubated at 37 °C with 5% CO_2_ and observed daily for seven days for the development of cytopathic effects.

For CoV isolation, cell cultures were used with growth media (Eagle’s minimum essential medium (EMEM)) supplemented with 10% fetal bovine plus antibiotics (100 units/ml of penicillin and 100 μg/ml of streptomycin), 0.3% tryptose phosphate broth (Sigma, USA), 0.02% yeast extract (Sigma, USA) and 10 μg/ml trypsin. Twenty-four well tissue culture plates were inoculated with 0.2 ml per well of the clarified pathological material. After adsorption for 1 h at 37 °C, maintenance medium EMEM supplemented with 1% fetal bovine serum and antibiotics (0.8 ml per well) was added without removing the viral inoculum, and the cultures were incubated at 37 °C.

### Whole-genome sequencing

Libraries were prepared following sequence independent single primer amplification (SISPA) with several variations as described by Djikeng et al. [17].

Nine microlitres of extracted RNA was used for reverse transcription reaction using a combination of random (FR26RV-N) and poly T (FR40RV-T) primers tagged with the sequence 5’-GCC GGA GCT CTG CAG ATA TC-3′, using SuperScript III Reverse Transcriptase (Invitrogen, Monza, Italy) following the manufacturer’s instructions.

The second strand of cDNA was synthesized by DNA Polymerase I Large (Klenow) Fragment (Promega, Milan, Italy) using 20 μl of cDNA. Twenty microlitres of Klenow product was amplified by the Expand High Fidelity PCR System (Sigma Aldrich S.R.L., Milan, Italy) using FR20RV-T primer complementary to the sequence tag. Five microlitres of the PCR product was analysed on a 1% agarose gel. The PCR amplicons were purified using the QIAquick PCR Purification Kit (Qiagen, Milan, Italy) following the manufacturer’s instructions and eluted in 40 μl of nuclease-free water.

The purified DNA was quantified in the Qubit 2.0 Fluorometer (Invitrogen) using the Qubit dsDNA HS Assay Kit (Thermo Fisher Scientific, Rodano, MI, Italy) following the manufacturer’s instructions. Five hundred nanograms of purified DNA were digested with EcoRV enzyme (New England BioLabs, Pero, MI, Italy) to remove the tag sequences. Digested DNA was cleaned up adding a 1.8× volume of Agencourt AMPure XP beads (Beckman, Milan, Italy). DNA was quantified by Quibit 2.0 Fluorometer and the library prepared by Ion Xpress Plus gDNA Fragment Library Preparation (Thermo Fisher Scientific) following the standard protocol for 100 ng of DNA. Emulsion PCR was performed using the Ion PGM Template OT2 200 Kit and the sequencing run performed ac-cording to the instructions of the manufacturer (Ion PGM Sequencing 200 Kit v2) (Thermo Fisher Scientific) by Ion Personal Genome Machine (PGM) in Ion 316 Chip v2.

### Phylogenetic analyses

Reads obtained by Ion Torrent sequencer were checked by quality control, cleaned up and trimmed using CLC Workbench version 5.5.1 (www.clcbio.com). A de novo assembly was performed using the default parameters and excluding contigs shorter than 1000 bases. Reads were mapped against the full genome using an online tool (Bowtie2, Galaxy Aries) and visualized by IGV software. MEGA7 was used to edit, align nucleotide and amino acid sequences and to calculate the pairwise identities of the genomes and all ORFs that were predicted using the online tool ORF Finder (NCBI, http://www.ncbi.nlm.nih.gov/gorf/gorf.html). Complete genome sequences of MERS-CoV and apha- and beta CoVs from bats, human and camels (n. 131) were obtained from the NIAID Virus Pathogen Database and Analysis Resource (ViPR) [18] through the web site at http://www.viprbrc.org/. Multiple sequence alignment was calculated using the MUSCLE algorithm. The maximum likelihood phylogenetic tree was performed using IQtree software [19] and Model finder to determine the best model according to BIC [20]. Genetic relationships between Italian and SA bats and MERS-CoV were confirmed by comparison of the sequence distances of MERS-CoV and bat-BCoV 2c (SA bat and IT bats) using SSE v1.2 [21]. The complete S protein and the S1 domain responsible for DPP4 recognition (located in a C-terminal 240 residue RBS of IT-bat CoVs) were compared with those of MERS-CoV, HKU4, HKU5 and MERS-related bat CoVs from China and Africa. To better investigate the relationship between MERS-CoV and related bat sequences, a maximum likelihood phylogenetic tree based on the S1 protein was constructed using the IQtree software including only beta CoV sequences. Similarity plots of CoV clade c genomes were generated by using SSE v1.2 using a sliding window of 600 and a step size of 100 nucleotides (nt).

### Three-dimensional macromolecular structure

The three dimensional macromolecular structure (3D–MMS) of the DPP4 binding domain in the S protein was predicted using the sequence-homology method that is based on sequences and structures released by the protein data bank (PDB) and visualized by Cn3D v4.3 software [22]. The secondary structure elements are defined based on an ESPript (http://espript.ibcp.fr) algorithm [23] and are labelled in a previous report on the MERS-RBD structure [24].

## Results

Two bat carcasses belonging to two different species, *Hypsugo savii* and *Pipistrellus kuhlii*, were provided in 2011 by a wildlife recovery centre located in the Modena province (North Italy)*.* The first bat was an adult female whereas for the second one data on age and sex were not available. During necropsy, no pathological lesions indicative of infectious diseases were observed in the two animals, but dehydration and traumatic injuries such as lacerations of the wing membrane were observed.

Samples of intestine from the two bats tested positive by the pan-coronavirus one-step RT-PCR method, and then cell cultures were inoculated with them to attempt virus isolation without success.

### Genome organization, phylogenetic and molecular analyses

Two complete genome sequences of Bat-CoV/Hypsugo savii/206645–40/2011 (BatCoV-Ita1) and Bat-CoV/Pipistrellus khulii/Italy/206645–63/2011 (BatCoV-Ita2) were obtained from total RNA extracted from portions of intestine. Comparison of the RdRp sequences of the two samples obtained by Sanger method by Lelli et al. [12] showed 99% nucleotide identity between them; Initial BLAST analysis revealed they were highly similar to the beta CoVs clade 2csequences. The two full genome sizes were 30,048 nt for BatCoV-Ita1 and 30,039 for BatCoV-Ita2, with a G + C content of 39% each.

The genome organization of BatCoV-Ita1 and BatCoV-Ita2 (IT bat CoVs) is identical to that of MERS CoV species encompassing the 10 open reading frames (ORFs) in the order of ORF1ab-spike-ORF3-ORF4ab-ORF5-envelope (E)-membrane (M)-nucleocapsid (N)-ORF8b and the common non-translated sequences identified in CoV genomes at the 5′ and 3′ genomic termini and between ORF5 and the E gene (Table 1).

**Table 1:** Genome localization of predicted protein sequences, putative leader TRS-L and TRS-B

BatCoV-Ita1			
ORF	nt position (start-end)	No. of amino acids	Sequence^a^
ORF1ab (TRS-L)	217–21,446	7076	_00036_GATTTTAACGAACTTAAA_00053_
Spike	21,388–25,425	1345	_21330_C.AG..........CGTT_21347_
ORF3	25,438–25,749	103	_25419_TCAC.A.....T.....T_25436_
ORF4a	25,758–26,045	95	_25742_A.AA..........CT.T_25759_
ORF4b	25,963–26,724	253	
ORF5	26,731–27,414	227	_26,_ _717_.G.GG.........ATGG_26734_
E	27,493–27,741	82	_27479_TTGGAA........ATGT_27496_
M	27,756–28,412	218	_27,734_.GG...........CTCT_27751_
N	28,460–29,749	429	_28,430_............TC.TT._28447_
ORF8b	28,506–29,084	192	
BatCoV-Ita2			
ORF	nt position (start-end)	No. of amino acids	Sequence^a^
ORF1ab (TRS-L)	208–21,437	7076	_00026_GATTTTAACGAACTTAAA_00043_
Spike	21,379–25,416	1345	_21321_C.AG..........CGTT_21338_
ORF3	25,429–25,740	103	_25410_TCAC.A.....T.....T_25427_
ORF4a	25,749–26,036	95	_25733_A.AA..........CT.T_25750_
ORF4b	25,954–26,715	253	
ORF5	26,722–27,405	227	_26,_ _708_.G.GG.........ATGG_26725_
E	27,484–27,732	82	_27470_TTGGAA........ATGT_27487_
M	27,747–28,403	218	_27,725_.GG...........CTCT_27742_
N	28,451–29,740	429	_28,421_............TC.TT._28438_
ORF8b	28,497–29,075	192	

^a^Dots represent identical nucleotides compared to the TRS-L

In ORF1ab the predicted slippery sequence “UUUAAAC” has been observed fitting the consensus motif X_XXY_YYZ (where XXX normally represents any three identical nucleotides; YYY represents strictly AAA or UUU; and Z represents A, C, or U) of nidoviruses involved in synthesis of the replicase pp1ab polyprotein by ribosomal frameshift.

**Fig. 1:**
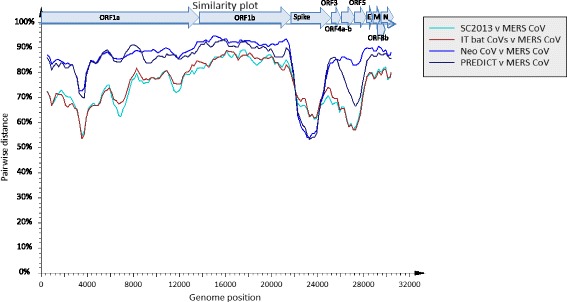
Sequence identity between IT bat CoVs and other prototype clade c betacoronaviruses and MERS related strains. Similarity plots were generated using SSE version 1.2 using a sliding window of 600 and a step size of 100 nucleotides

The size and genomic localization of the nonstructural protein (NSP 1–16) encoded by ORF1ab were predicted by sequence comparison with other beta CoV species. Table 2 shows the 15 expected cleavage sites, 11 recognized by the “main protease” 3C–like protease (3CLpro, NSP4–10, NSP12–16), 3 by papain-like protease (PL2pro, NSP1–3) as well as the autocatalytic site (NSP11). The IT bat CoV cleavage sites, recognized by viral proteases, were identical to those of a BatCoV isolated in China (BtVs BetaCoV/SC2013) and differed from the MERS-CoV by one amino acid in the cleavage sites between NSP1/2 and NSP6/7 (Table 3). A predicted leader transcription regulatory sequence (TRS-L), and seven putative transcription regulatory sequences body TRS-B, representing signals for the discontinuous transcription of subgenomic mRNAs (sgmRNAs), have been identified. The two IT bat isolates shared the same TRS-L, the seven TRS-B (Table 1) as well as 98.8% nucleotide identity. Across the whole genome, the percentage of overall nucleotide identity among other beta CoVs was 80% to MERS CoV, 82% to SC2013, 82% to NeoCoV, 81% to PREDICT, 72.4% to bat CoVs HKU4 and 72,5% to HKU5. The genomic sequence identity between IT bat CoVs, MERS and other MERS-related Bat CoVs is reported in Fig. 1; in particular the lowest identity of the IT bat CoVs with the other beta CoV strains was evidenced in the genomic regions encoding Pl2pro (NSP3) within ORF1ab, Spike, ORF4ab and ORF5.

**Table 2:** Prediction of the putative pp1a/pp1b cleavage sites of BatCoV-Ita1/2 based on sequence comparison with MERS-CoV strain HCoV-EMC/2012

NSP	Position of the putative cleavage sites^a^	Protein size (no. of amino acids)	Putative functional domain(s)^b^
NSP1	Met^1^-Gly^195^	195	
NSP2	Asn^196^-Gly^855^	660	
NSP3	Ala^856^-Gly^2738^	1883	ADRP, PL2pro
NSP4	Ala^2739^-Gln^3245^	507	
NSP5	Ser^3246^-Gln^3551^	306	3CLpro
NSP6	Ser^3552^-Gln^3843^	292	
NSP7	Ser^3844^-Gln^3926^	83	
NSP8	Ala^3927^-Gln^4125^	199	Primase
NSP9	Asn^4126^-Gln^4235^	110	
NSP10	Ala^4236^-Gln^4375^	140	
NSP11	Ser^4376^-Ile^4389^	14	Short peptide at the end of *ORF1a*
NSP12	Ser^4376^-Gln^5309^	934	RdRp
NSP13	Ala^5310^-Gln^5907^	598	HEL, NTPase
NSP14	Ser^5908^-Gln^6431^	524	ExoN, NMT
NSP15	Gly^6432^-Gln^6773^	342	NendoU
NSP16	Ala^6774^-His^7076^	303	OMT

^a^Superscript numbers indicate positions in polyprotein pp1a/pp1ab or position in available sequence with the supposition of a ribosomal frameshift based on the conserved slippery sequenced (UUUAAAC) of Coronaviruses. Localized at nucleotide position 13,359–13,365 for BatCoV-Ita1 and 13,350–13,356 for BatCoV-Ita2

^b^ *ADRP* ADP-ribose 1-phosphatase, *PL2pro* papain-like protease 2, *3CLpro* coronavirus NSP5 protease, *Hel* helicase, *NTPase* nucleoside triphosphatase, *ExoN* exoribonuclease, *NMT* N7 methyltransferase, *NendoU* endoribonuclease, *OMT* 2’ O-methyltransferase

**Table 3:** Comparison of the predicted pp1a/pp1b cleavage site sequences^a^ of BatCoV-Ita1/2 with prototype clade c betacoronaviruses and MERS related strains

	NSP1	NSP2	NSP3	NSP4	NSP5	NSP6	NSP7	NSP8	NSP9	NSP10	NSP11	NSP12	NSP13	NSP14	NSP15
BatCoV-Ita1/2	LVGG	LKGG	IVGG	LQS	MQS	VQS	LQA	LQN	LQA	TQS	RGSI	LQA	LQS	VQG	LQA
MERS^b^	-I--	–	–	–	–	M--	–	–	–	P--	–	–	–	–	–
HKU4^c^	-I--	–	–	–	–	–	–	–	–	P--	GS-V	–	–	–	–
HKU5^d^	–	–	LS--	–	–	–	–	–	–	P--	–	–	–	I--	–
Erinaceus^e^	-C--	–	–	–	–	–	--S	–	–	LH-	–	–	–	–	–
NeoCoV^f^	-T--	–	–	–	–	I--	–	–	–	P--	–	–	–	–	–
BtVs-BetaCoV/SC2013^g^	–	–	–	–	–	–	–	–	–	P--	–	–	–	–	–

^a^Hyphens represent identical amino acids compared to the BatCoV-Ita1/2 sequences

^b^GenBank accession number JX869059

^c^GenBank accession number EF065505

^d^GenBank accession number EF065509

^e^GenBank accession number KC545386

^f^GenBank accession number KC869678

^g^GenBank accession number KJ473821

**Fig. 2:**
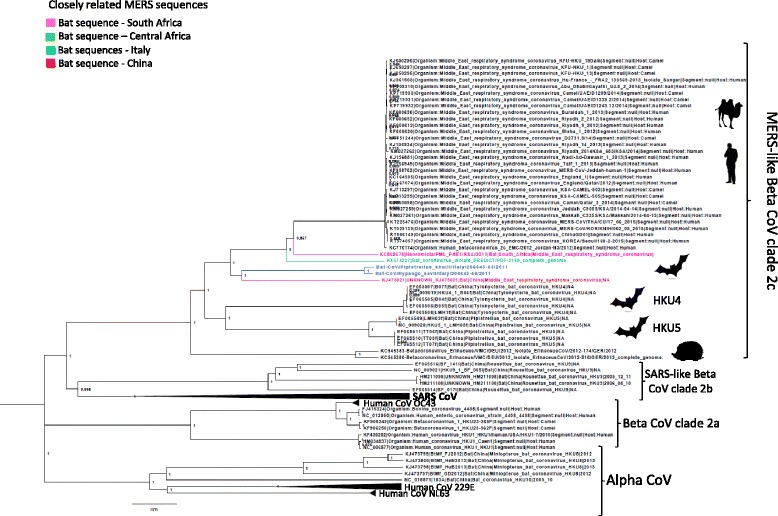
Maximum phylogenetic tree based on alpha and beta CoV full genomes. Ultrafast bootstrap approximation approach was performed to compute the support of phylogenetic groups. Best-fit model according to BIC was GTR + G4. Bat sequences close related to MERS-CoVs are reported in colors: light blue for IT bat CoVs, pink for NeoCoV from South Africa, green for PREDICT from Uganda and red for SC2013 from China. Bat-CoV/H.savii/Italy/206645–40/2011 and Bat-CoV/P.khulii/Italy/206645–63/2011 sequences can be retrieved under accession numbers MG596802 and MG596803 respectively

A phylogenetic tree of the complete genomes showed a monophyletic group placed in the Beta2c clade formed by MERS-CoV sequences originating from humans and camels and bat-related sequences. The closest bat sequences are those originating from Africa, Italy and China (Fig. 2).

Comparison of the predicted protein sequences of the IT bat CoVs and the other beta CoVs showed the highest amino acid sequence identities for E (69.5–91.5%), M (81.7–83.7) and N (71.8–83.7%) proteins and the lowest in ORF3 (28.6–53.5%) and ORF 4b (29.9–56%)(Table 4). Comparison of MERS-CoV protein sequences to EriCoV, HKU4 or HKU5 displayed the highest amino acid sequence identities in the E, M and N proteins whereas the lowest were observed in the S and ORF4b.. In particular, the S protein of MERS-CoV showed above 50% identity to the related bat S proteins: 68.8% to IT bat CoVs, 68.6% to SC2013, 67.7% to HKU4, 64.9% to HKU5 and 64% to NeoCoV. However, if we analyse only the RBD region spanning amino acids 367–606 within the S1 subunit, which is the DPP4-interacting region, the percentage of identity to MERS changed to being slightly higher (54.1–55.0%) for HKU4 with respect to other CoVs (HKU5, IT-batCoVs) (52.5–51.7%). The RBD of Neo CoV showed the lowest percentage of identity to MERS (33.5%).

**Table 4:** Comparison between predicted protein sequences of the IT bat CoVs and prototype clade c betacoronaviruses and MERS related strains

	% Amino acid identities^a^						
ORF	IT Bat CoVs	MERS-CoV^b^	HKU4^c^	HKU5^d^	EriCoV^e^	NeoCov^f^	BtVs-BetaCoV/SC2013^g^
ORF1ab	99.4	81.3-81.5	73.5–73.6	75.9–76	74.5–74.7	81.6	84.6–84.7
Spike	99	68.5–68.8	70.4–70.6	73.8–74.3	57.8–58.1	60.9	79.6
ORF3	97.1	48.5–49.5	39.6–40.7	46.5–47.5	28.6	51.5	53.4
ORF4a	97.9	53.7	44.2	50–52.2	43.2–43.4	54.7	67
ORF4b	98	43.9–46.4	29.9–30.3	31.1–32	39.9–40.4	47.4	56
ORF5	98.7	64.3–64.7	47.6	54.5–55	52.9	62.9	74
E	100	86.6	70.7	69.5	78	91.5	91.5
M	99.5	84.9–85.8	81.7–82.1	82.1–82.6	83.9–84.4	84.9	87.2
N	99.5	81.4–81.9	73–73.4	71.8	74.4–74.6	83.7	83.4
ORF8b	96.9	63.4–67	49.2–52.4	49.5–52.1	47.9–49.5	63–65.1	64–65.6
Concatenated domains	99.3	79	74	74.6	74.4	79.2	82.3

^a^Calculated with MEGA7 using a pairwise deletion option

^b^GenBank accession number JX869059, KC164505, KC776174, KF186567, KF192507, KF600612, KF600620, KJ477102

^c^GenBank accession number EF065505, EF065506, EF065507, EF065508, DQ648794

^d^GenBank accession number EF065509, EF065510, EF065511, EF065512

^e^GenBank accession number KC545386, KC545383

^f^GenBank accession number KC869678

^g^GenBank accession number KJ473821

**Fig. 3:**
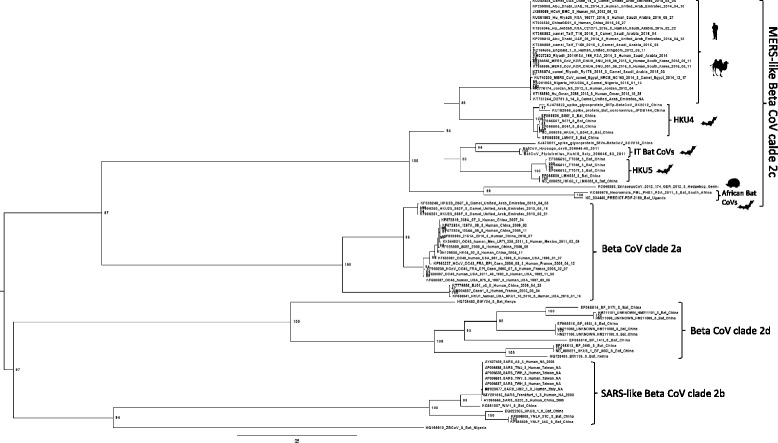
Maximum phylogenetic tree based on deduced amino acid sequences of S1 protein of beta CoVs. Best-fit model according to BIC was WAG + F + I + G4. Clades 2a, 2b, 2c and 2d corresponding to the sequences of human CoVs OC43, SARS-like, MERS-like and HKU9 CoV respectively are identified

Moreover, the phylogenetic tree based on the S1 protein (Fig. 3) shows four clearly different clades: 2a, 2b, 2c and 2d corresponding to the sequences of human CoVs OC43, SARS-like, MERS-like and HKU9 CoV, respectively. Clade 2c reflects the RBD percentage of identity and was further differentiated into three groups: MERS sequences and the highly related HKU4 sequences form one group; HKU5 and IT-batCoVs sequences were placed in the second group; the third one, which is the most distant, includes African bats and hedgehog sequences.

**Fig. 4:**
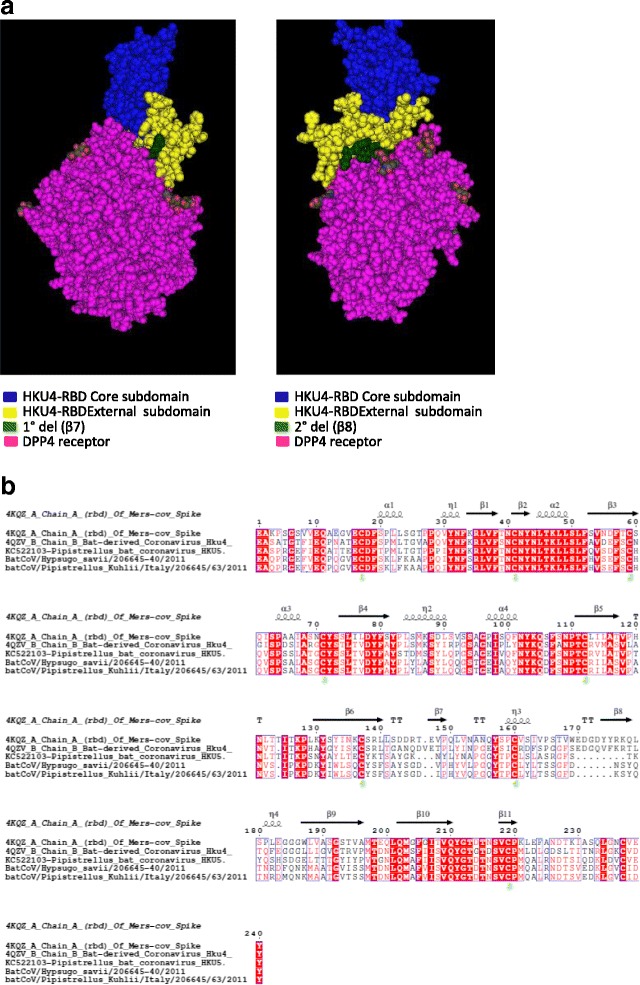
Comparison of secondary and tertiary structures of MERS-like CoV RBD. **a** Predicted 3D MMS of the core and external subdomains of HKU4 RBD and human DPP4 (right and left lateral view). The two deletions observed in IT Bat CoVs RBD are evidenced in green. **b** Structure –based sequence alignment . The secondary structure elements are defined based on an ESPript algorithm and are labeled as in a previous report on the MERS RBD structure [24]. Spiral lines indicate helices, while arrows represent β strands. The external subdomain is highlighted by enclosure in a blue box. The two deletions found in IT Bat CoVs and HKU5 RBD are marked with blue lines. The Arabic numerals 1–4 indicate cysteine residues that pair to form disulfide bonds

The secondary and three dimensional structures of the RBD domain of IT bat CoVs were analysed in comparison with the MERS, HKU4 and HKU5 sequences. Two aa deletions located in a region corresponding to the external subdomain of MERS-RBD were found in IT bat CoV-RBDs in the same positions as in HKU5-RBD: three and eight aa in the HKU5-RBD and two and six in IT bat CoVs. The two deletions are located in two regions corresponding to scaffold strands β7 and β8 in the MERS/HKU4-RDB structure (Fig.4a, b). These two β strands together with β6 and 9 form the external subdomain characterized by four anti-parallel β strands that expose a flat sheet-face for receptor engagement [8].

## Discussion

The high diversity of bat species as well as other unique biological and ecological features, such as long life span, roosting, migratory behaviour and the use of torpor and hibernation, contributes to bats being considered natural hosts of a large number of diverse viruses [25]. Another important characteristic is the evolution of flight, which is the most peculiar characteristic of bats and one of the most important for their wide distribution; it may have had effects on some aspects of the evolution of the immune system and the metabolism of bats and could allow them to host different viruses [26, 27]. Bats are also demonstrated natural reservoirs of many alpha CoVs and beta CoVs, which provide viral genes for the genesis of newly emerging coronaviruses with interspecies transmission potential.

Because of its similarity to the SARS CoV, it had been proposed that bats were somehow involved in transmission of the MERS CoV. Indeed, Memish et al. [5] detected a partial RNA sequence of a beta CoV obtained from a faecal pellet from an Egyptian tomb bat that showed 100% identity to the virus from the human index case-patient. The emergence of MERS CoV probably involved genetic exchanges between different viral ancestors that may have occurred either in bat ancestors or in camels acting as mixing vessels for viruses from different hosts. Recent studies have suggested that one-humped camels (*Camelus dromedarius*) may be a primary source of this virus in nature [28], and experimental infections of camels with MERS CoV seem to support this view [29].

In this study, the full genomes of two beta CoVs closely related to MERS obtained from two Italian bats belonging to the *P. kuhlii* and *H. savii* species are reported. Italy is an area of high bat species diversity with more than 30 bat species documented by historical records and recent studies, but these two species, which belong to Vespertionilidae family, are the most frequently recorded [30]. *Pipistrellus kuhlii* forages over a variety of habitats, including agricultural and urban areas (including around street lights). Recent evidence suggests that urbanization may be beneficial to this species in that colonies in urban and suburban areas have advanced parturition and produce more offspring than colonies in rural areas, at least in central Italy [31]. *H. savii* forages over open woodland, pasture and wetlands and often feeds at lights in rural areas, towns and cities. This is one of the most common species in the Italian Mountains, the Apennines and the Alps below 2600 m.

Detection of viruses belonging to clade 2c seems to be particularly associated with vespertilionid bats even if this association is not exclusive. Indeed, NeoCoV, BtVs-BetaCoV/SC2013, PREDICT/PDF-2180, HKU4, HKU5 and the two IT bat CoVs were all found in species belonging this family.

The full IT bat CoV sequences were obtained from two bat carcasses and showed the same genome organization as MERS-CoV either for the 10 open reading frames (ORFs) or the common non-translated sequences identified in CoV genomes. The overall nucleotide identity to MERS CoV is close to 78%, and in the phylogenetic tree they are represented in the same MERS-like clade 2c. From the molecular point of view, the International Committee on Taxonomy of Viruses (ICTV) has established 90% amino acid sequence identity as the threshold value for CoV species demarcation of the seven concatenated domains within the ORF1ab: NSP3 (ADRP), NSP5 (3CLpro), NSP12 (RdRp), NSP13 (Hel, NTPase), NSP14 (ExoN, NMT), NSP15 (NendoU), NSP16 (OMT). The sequence identity of the BatCoV-Ita1 and BatCoV-Ita2 concatenated domains is below the threshold value compared to HKU4, HKU5 and EriCoV (86.1–89.2%) and over the threshold value compared to MERS-CoV, NeoCoV and BtVs-BetaCoV/SC2013 (92–92.9%), indicating that the two IT bat CoVs could be included in the same virus species as MERS-CoV and related isolates.

Full genome phylogenetic reconstruction showed that the two African bat-CoV sequences were the ones most closely related to MERS; however, the spike gene evidenced higher sequence differences with respect to HKU4 and other related bat sequences, IT bat CoVs included. Indeed, it was demonstrated that the RBD domain in the S1 protein of HKU4 but not HKU5 can bind to the human DPP4 receptor even if with less affinity. The marked difference between HKU4-RBD and HKU5-RBD with respect to MERS-RBD is the presence of two marked deletions in the external subdomain responsible for receptor recognition [24]. Two similar deletions in the region corresponding to scaffold strands β7 and β8 in the MERS/HKU4-RBD structure were observed in IT bat CoVs, suggesting also for them the absence of DPP4-binding potential. Based on these results we can hypothesize that human DPP4 is not a functional receptor for IT bat CoVs as previously shown for HKU5-CoVs.

## Conclusions

The role played by bats in the maintenance and transmission of beta CoVs, if they are simply incidental hosts or competent reservoir hosts able to transmit them to other vertebrates, is an open question that must be carefully addressed. It is believed that the majority of all alpha and beta CoVs currently circulating in mammals are evolutionarily linked to ancestral CoVs originated from bats [4]. However, more surveillance studies are needed to better investigate the potential intermediate hosts that may play a role in the interspecies transmission of known and currently unknown coronaviruses. Particular attention should be paid to investigating the S protein sequences and structures as well as receptor specificity and binding affinity as keys to understanding the biology of bat-derived viruses, their potential threat to human health and the evolutionary pathway of MERS-CoV.

## Abbreviations

3DMMS: Three dimensional macromolecular structure
CoV: Coronavirus
DPP4: Dipeptidyl peptidase 4
ICTV: International Committee on Taxonomy of Viruses
MERS-CoV: Middle Est respiratory syndrome coronavirus
PDB: Protein data bank
RBD: Receptor binding domain
SARS-CoV: Severe acute respiratory syndrome coronavirus
TRS-B: Transcription regulatory sequence body
TRS-L: Transcription regulatory sequence leader
