# Correction to: Detection and full genome characterization of two beta CoV viruses related to Middle East respiratory syndrome from bats in Italy

**DOI:** 10.1186/s12985-018-0921-y

**Published:** 2018-01-12

**Authors:** Ana Moreno, Davide Lelli, Luca De Sabato, Guendalina Zaccaria, Arianna Boni, Enrica Sozzi, Alice Prosperi, Antonio Lavazza, Eleonora Cella, Maria Rita Castrucci, Massimo Ciccozzi, Gabriele Vaccari

**Affiliations:** 1 Department of Virology, Istituto Zooprofilattico Sperimentale Lombardia ed. Emilia Romagna, Via Antonio Bianchi 9, 25124 Brescia, Italy; 2 Department of Food Safety, Nutrition and Veterinary Public Health, Istituto Superiore di Sanità., Viale Regina Elena 299, 00161 Rome, Italy; 3 Dept. of Sciences, University Roma Tre, Viale Guglielmo Marconi 446, 00146 Rome, Italy; 4 University Campus Bio-Medico of Rome, Via Álvaro del Portillo, 21, 00128 Rome, Italy

## Correction

After Publication of the article [1], it has been brought to our attention that an author’s name has been spelt incorrectly. The correct spelling should be “Massimo Ciccozzi”, but it was previously included as “Massimo Cicozzi”. The original version has now been revised to reflect this.
